# Forkhead Box C1 Regulates Human Primary Keratinocyte Terminal Differentiation

**DOI:** 10.1371/journal.pone.0167392

**Published:** 2016-12-01

**Authors:** Lianghua Bin, Liehua Deng, Hengwen Yang, Leqing Zhu, Xiao Wang, Michael G. Edwards, Brittany Richers, Donald Y. M. Leung

**Affiliations:** 1 The First Affiliated Hospital, Biomedical Translational Research Institute, the International Immunology Center and the Key Laboratory of Antibody Engineering of Guangdong Province, Jinan University, Guangzhou, Guangdong Province, China; 2 Department of Pediatrics, National Jewish Health, Denver, Colorado, United States of America; 3 Division of Pulmonary Sciences and Critical Care Medicine, Department of Medicine, University of Colorado Denver, Aurora, CO, United States of America; 4 Guangdong Provincial Key Laboratory of Allergy & Clinical Immunology, The State Key Clinical Specialty in Allergy, The Second Affiliated Hospital of Guangzhou Medical University, Guangzhou, Guangdong Province, China; Feinberg Cardiovascular Research Institute, Northwestern University, UNITED STATES

## Abstract

The epidermis serves as a critical protective barrier between the internal and external environment of the human body. Its remarkable barrier function is established through the keratinocyte (KC) terminal differentiation program. The transcription factors specifically regulating terminal differentiation remain largely unknown. Using a RNA-sequencing (RNA-seq) profiling approach, we found that forkhead box c 1 (FOXC1) was significantly up-regulated in human normal primary KC during the course of differentiation. This observation was validated in human normal primary KC from several different donors and human skin biopsies. Silencing FOXC1 in human normal primary KC undergoing differentiation led to significant down-regulation of late terminal differentiation genes markers including epidermal differentiation complex genes, keratinization genes, sphingolipid/ceramide metabolic process genes and epidermal specific cell-cell adhesion genes. We further demonstrated that FOXC1 works down-stream of ZNF750 and KLF4, and upstream of GRHL3. Thus, this study defines FOXC1 as a regulator specific for KC terminal differentiation and establishes its potential position in the genetic regulatory network.

## Introduction

Epidermal keratinocyte (KC) terminal differentiation is essential for the acquisition of the normal skin barrier function and homeostasis [1]. Aberrant barrier function is associated with various skin diseases, including atopic dermatitis, psoriasis, ichthyosis vulgaris, etc [2,3]. Several transcription factors have been identified to regulate the late differentiation and barrier formation, including KLF4, ZNF750, GATA3, GRHL3, and PRDM1 (Blimp-1)[4–8]. Recently, two additional transcription factors, MAF and MAFB, were identified as important KC terminal differentiation regulators [9]. MAF and MAFB working together to regulate KLF4, ZNF750, GRHL3 and PRDM1. These studies suggest there is a regulatory network of transcription factors involved in KC terminal differentiation. Due to the high complexity of this regulatory program, it is likely that additional important regulators in this process remain to be identified.

Through an exploratory gene profiling analyses of human normal primary KC during the progression of calcium-induced differentiation, we found that Forkhead box C1 (FOXC1) is significantly up-regulated in differentiated KC (GSE 73305). *FOXC1* is a member of the forkhead box transcription factors. These proteins are distinguished by a characteristic 100-amino acid DNA-binding motif that is highly homologous to Drosophila forkhead genes. FOX proteins are critical regulators in the development of tissue differentiation, embryogenesis and tumorigenesis [10]. Here, in this study, we first validate the discovery from our gene profiling analyses that FOXC1 is induced in differentiated KC in cultured human primary KC and human skin biopsies. We further demonstrate that FOXC1 is necessary for human KC terminal differentiation through inhibiting FOXC1 gene expression and FOXC1 over-expression experiments. In the KC terminal differentiation regulatory network, FOXC1 is a target gene of ZNF750 and KLF4, and works up-stream of GRHL3.

## Results

### FOXC1 is up-regulated in differentiated human primary KC

To discover novel effectors for human KC differentiation, we profiled gene expression of undifferentiated human KC and differentiated KC under Ca^2+^ -induced differentiation over a 5-day time course (S1 Appendix). The gene profile data have been deposited in the NCBI Gene Expression Omnibus (access number: GSE 73305). We searched for previously uncharacterized transcription factors that were significantly up-regulated during the course of KC differentiation. This effort led us to find that FOXC1 is significantly up-regulated during the progression of KC differentiation (S1 Table).

**Fig 1:**
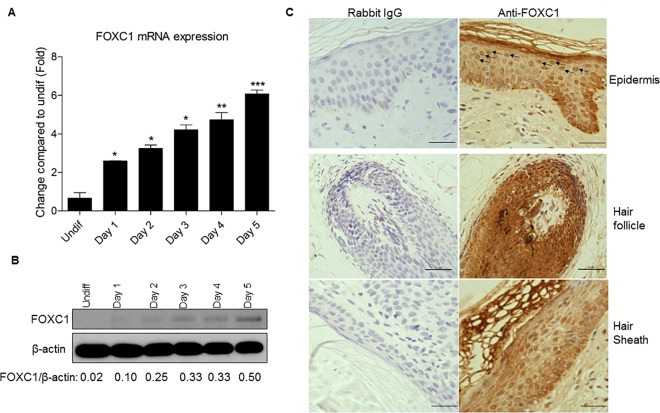
FOXC1 is induced in differentiated keratinocytes. A) mRNA levels of FOXC1 is increasing during the course of KC differentiation. Data presented as mean ±SEB. “undiff” is the abbreviation of undifferentiated KC. * p<0.05; ** p<0.01; *** p<0.001. The data are representative of four independent experiments. B) FOXC1 protein is increasing along with KC differentiation degree. The data represent one of the three independent experiments. C) Immunohistochemistry staining of FOXC1 protein expression in human epidermis, hair follicle and the differentiated inner hair root sheath. FOXC1 positive staining is presented as brown color in cell nuclear (Scale bar, 50 μm). The arrows point to examples of typical positive staining.

We first validated the results by real-time PCR and western blots using human primary keratinocytes from different human donors. The cells were differentiated by elevation of calcium concentration in culture media up to 5 days. As shown in Fig 1A, the mRNA of FOXC1 was significantly up-regulated during the course of KC differentiation, and its protein expression was also gradually increased in differentiated KC (Fig 1B). A recent paper reported that FOXC1 is expressed in mouse hair follicles but absent in mouse epidermis [11]. In our study, however, we find that FOXC1 is expressed not only in human hair follicles, but also in the differentiated KC of inner hair root sheath that surrounds the hair shaft and the epidermis upper layers of KC (Fig 1C). These data provide *in vivo* evidence that FOXC1 is associated with KC differentiation in humans.

### Up-regulation of FOXC1 is necessary for KC terminal differentiation

**Fig 2:**
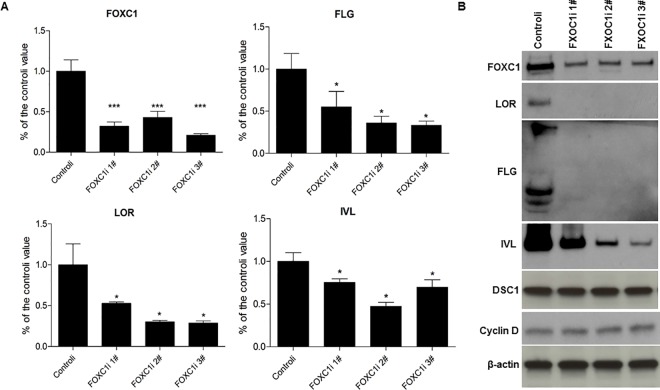
Silencing FOXC1 leads to deceased gene expression of KC differentiation markers. A) mRNA expression of FLG, IVL and LOR in FOXC1 silenced differentiated KC and control cells evaluated by real-time PCR. Data represented as mean ±SEB. The data are representative of four independent experiments. B) Protein expression of FLG, IVL, LOR, DSC1 and Cyclin D in FOXC1 silenced differentiated KC and control cells evaluated by western -blot. The data represent one of the three independent experiments.

**Fig 3:**
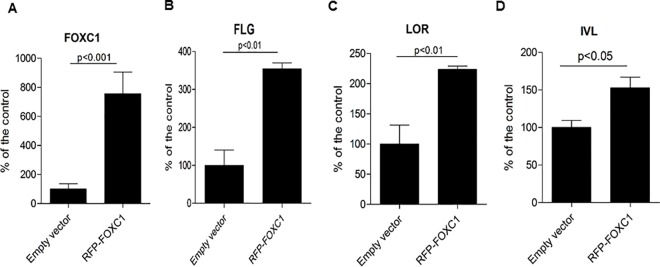
Over-expression of FOXC1 leads to increased gene expression of KC differentiation markers. mRNA expression of FLG, IVL and LOR in FOXC1 over-expressed KC and control cells evaluated by real-time PCR. Data represented as mean ±SEB. The data are representative of three independent experiments.

To test for a potential functional role for FOXC1 in KC terminal differentiation, FOXC1 siRNA (FOXC1i) and scrambled siRNA (Controli) duplexes were transfected into undifferentiated KC. The cells were then allowed to undergo Ca2+ -induced differentiation. Although the early differentiation markers keratin 1 and 10 were unaffected (data not shown), silencing FOXC1 resulted in reduced induction of late differentiation genes, including filaggrin (FLG), involucrin (IVL), and loricrin (LOR) in both mRNA and protein levels (Fig 2A & 2B). However, loss of FOXC1did not change the protein expression of desmocollin 1 (DSC1), a desmosome cell-cell adhesion protein (Fig 2B). Inhibition of FOXC1gene expression did not change cyclin D protein levels as compared to the control cells in Ca^2+^-driven differentiated KC. We also examined and compared the cell cycle parameters of undifferentiated KC transfected with scrambled siRNA and FOXC1 siRNA, and found no difference between normal cells and FOXC1 deficient KC (S1 Fig). These data suggest that FOXC1 doesn’t change the cell cycle of undifferentiated and differentiated KC. We also over-expressed FOXC1 in proliferative undifferentiated KC. In contrast to loss of expression, over-expression of FOXC1 led to increased mRNA levels of FLG, LOR and IVL in the absence of Ca^2+^ induction (Fig 3). Due to technical difficulty of growing primary human KC on glass cover slides for immunostaining, we couldn’t test the protein expression of FLG, LOR and IVL in the same cells with FOXC1 over-expression. Nevertheless, these data along with FOXC1 positive staining in human epidermis upper layer KC demonstrate that FOXC1 plays a role in KC terminal differentiation.

### Transcriptome Analysis of FOXC1-regulated Genes

**Fig 4:**
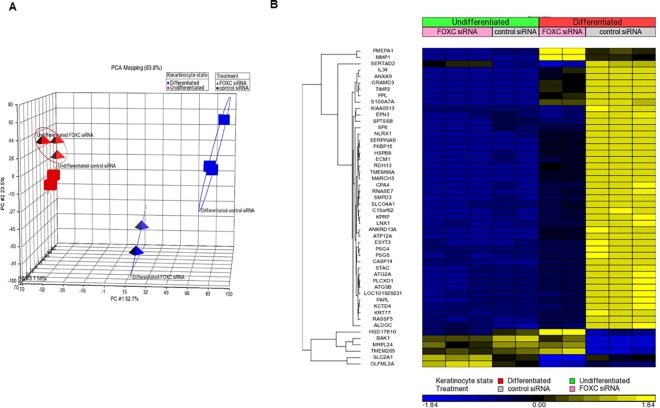
Transcription profiles of FOXC1 silenced KC are significantly different from control cells upon differentiation. A) A principle component assay shows separation of differentiated FOXC1 silenced KC from control cells. B) Heatmap shows the top 50 most differentially expressed genes in FOXC1 silenced undifferentiated and differentiated KC as compared to scrambled siRNA silenced undifferentiated and differentiated KC (FDR<0.01).

To identify the specific FOXC1 targeting genes among the global gene sets identified in KC differentiation, we profiled FOXC1 silenced KC versus control using a RNA-seq approach. We plotted the transcriptional data in three-dimensional space using principal component analysis (PCA) as implemented by Partek Genomics Suite using a standard correlation method, observing clear separation of FOXC1 silenced KC and control KC after differentiation (Fig 4A). In contrast, loss of FOXC1 expression in undifferentiated KC did not separate significantly from control cells.

Down-regulated genes in FOXC1 siRNA treated cells represent targets that are positively regulated by FOXC1. Among the top 50 most significantly differentially expressed genes across the four experimental groups (False discovery rate <0.01), 42 genes were down-regulated in FOXC1 silenced differentiated KC (Fig 4B), including KC terminal differentiation markers of periplakin (PPL)[12], keratinocyte proline-rich protein (KPRP)[13] and Epsin 3 (EPN3)[14], lipids synthesis genes of serine palmitoyltransferase (SPTSSB) and sphingomyelin phosphodiesterase 3 (SMPD3) and FLG maturation related protease gene caspase 14 (CASP14)[15–17]. Additionally, the majority of genes located at epidermal differentiation complex (EDC) of 1p21 [18]were decreased in differentiated KC upon FOXC1 silencing (Table 1). Aside from the EDC genes, kallikrein genes, that are important proteases functioning in desquamation and skin barrier homeostasis [19,20], are targets positively regulated by FOXC1 (Table 1). ABCA12, a gene with function in keratinocyte lamellar body lipid transportation and its loss-of-function mutations are associated with harlequin ichthyosis [21–23], was also significantly reduced in FOXC1-silenced KC (Table 1). The identity of FOXC1-regulated genes is consistent with a crucial role for FOXC1 in promoting KC differentiation.

**Table 1:** Down-regulated Keratinocytes differentiation markers in FOXC1 silenced KC.

LCE family		SPRR family		S100 fuse genes		KLK family		Lipid synthesis and transport and other gene	
Gene-Symbol	Log2 Ratio*	Gene-Symbol	Log2 Ratio*	Gene-Symbol	Log2 Ratio*	Gene-Symbol	Log2 Ratio*	Gene-Symbol	Log2 Ratio*
LCE1A	-2.21	SPRR2G	-1.25	FLG	-3.43	KLK10	-1.09	ABCA12	-1.25
LCE1B	-2.94	SPRR3	-1.70	FLG2	-4.70	KLK12	-0.72	ACER1	-1.33
LCE1C	-2.52	SPRR4	-2.93	TCHH	-0.86	KLK13	-1.30	ACER2	-1.73
LCE1D	-2.35			TCHHL1	-2.84	KLK14	-1.07	SMPD3	-2.28
LCE1E	-2.41			RPTN	-2.74	KLK4	-1.32	SPTSSB	-2.50
LCE1F	-2.35			CRNN	-4.76	KLK5	-0.95		
LCE2A	-1.58			HRNR	-1.47	KLK6	-1.80		
LCE2B	-2.05					KLK7	-0.90		
LCE2C	-1.57					KLK9	-3.80		
LCE2D	-2.04								
LCE3C	-3.65								
LCE3D	-1.70								
LCE3E	-2.10								
LCE5A	-2.27								
LCE6A	-2.60								

*Gene expression ratio in FOXC1 silenced differentiated KC versus in Scrambled siRNA silenced differentiated KC.

### The hierarchy role of FOXC1 in KC regulatory network

**Fig 5:**
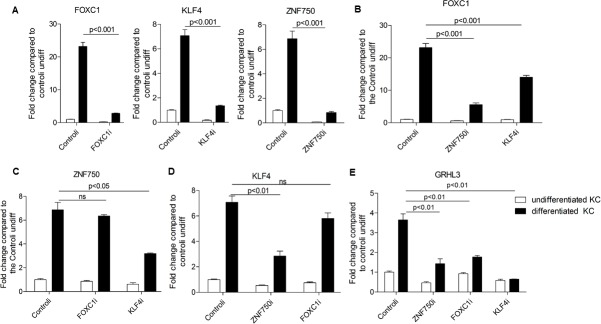
FOXC1 acts down-stream of ZNF750 and KLF4, and up-stream of GRHL3. A) FOXC1 expression in ZNF750 and KLF4 silenced KC. B) ZNF750 expression in FOXC1 and KLF4 silenced KC. C) KLF4 expression in ZNF750 and FOXC1 silenced KC. D) GRHL3 expression in ZNF750, KLF4 and FOXC1 silenced KC. Gene expression was evaluated by real time PCR. Data represented as mean ±SEB. The data are representative of three independent experiments.

ZNF750 and KLF4 are target genes of P63 and MAF/MAFB, and they are also essential regulators of epidermal differentiation [5,9]. To determine the hierarchy cascade of ZNF750, KLF4 and FOXC1, we knocked-down ZNF750 and KLF4 in KC progenitor cells that were then induced to differentiation. As shown in Fig 5A, FOXC1, KLF4 and ZNF750 were significantly inhibited by siRNA silencing. As compared to control cells, FOXC1 gene expression was significantly down-regulated in ZNF750 and KLF4 silenced KC (Fig 5B). In contrast, gene expression of ZNF750 and KLF4 were not affected by FOXC1 knockdown (Fig 5C & 5D). We found that ZNF750 silencing led to reduced gene expression of KLF4, reciprocally, KLF4 silencing resulted in reduced gene expression of ZNF750 ((Fig 5C & 5D). Additionally, the gene expression of GRHL3 was down-regulated in ZNF750, KLF4 and FOXC1-silenced KC (Fig 5E). These results demonstrated that FOXC1 acts down-stream of ZNF750 and KLF4, up-stream of GRHL3, and ZNF750 and KLF4 reciprocally regulate each other.

**Fig 6:**
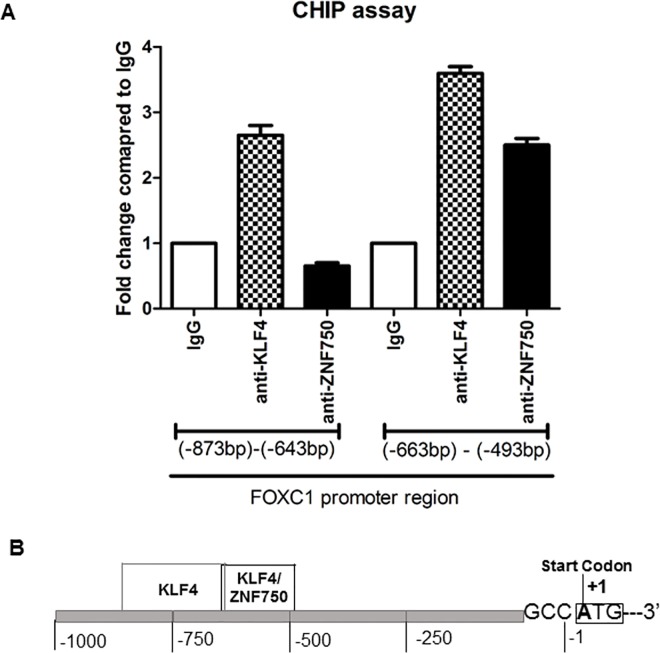
ZNF750 and KLF4 bind to the promoter region of FOXC1 gene. A) ChIP assays were performed using Ca^2+^ -driven differentiated keratinocytes. 9 pairs of primers covering 2 kilobase region of the upstream of FOXC1 start codon. Increased binding activity was found in the regions of -873bp to -643bp and -663bp to -493bp up-stream of the start codon. Data represented as mean ±SEB. B) The Schematic diagram of FOXC1 promoter region. KLF4 and ZNF750 binding sites are indicated.

To further validate whether FOXC1 is regulated by ZNF750 and KLF4 in keratinocytes, we used chromatin immune-precipitation (ChIP) to determine whether ZNF750 and KLF4 protein interact with FOXC1 promoter. We found that both ZNF750 and KLF4 bind to the promoter region of FOXC1: KLF 4 binds to two regions of -873bp to -643 bp and -663bp to -493 bp upstream of the start codon; whereas ZNF750 only binds to the -663bp to -493bp region up-stream of the FOXC1 start codon (Fig 6).

## Discussion

In our current study, we defined a previously unidentified role for the transcription factor FOXC1 in regulating the differentiation gene program in KC. Through siRNA knockdown experiments and transcriptome analysis as well as over-expression experiments, plus real-time PCR and western-blot validation, we demonstrated that FOXC1 activates an array of genes representing the terminal differentiation stage of human epidermal KC. Although a previous large scale multi-technique study of KC terminal differentiation mentioned the increased mRNA expression of FOXC1 in the granular layer of KC in human epidermis [24], our study is the first to demonstrate the regulatory function of FOXC1 in human somatic epidermal KC differentiation. FOXC1 knockout mice die prenatally with hydrocephalus, eye defects, and multiple skeletal abnormalities, demonstrating its critical role in the development of these tissues and organs [25]. Its function, however, in epidermal differentiation after birth has never been studied. Although FOXC1 mutations were found in human Axenfeld-Rieger syndrome which has no skin barrier disorder, the Axenfeld-Rieger syndrome is resulted from FOXC1 haploinsufficiency, which may not functionally damage enough to cause epidermal symptoms. Recently, while we were revising our submission, Cui et al published elegant work on the Journal of Investigative Dermatology reporting that specifically disrupted FOXC1 gene expression in mouse sweat gland epithelium led to symptoms of hypohidrosis; loss of FOXC1 in mouse sweat gland epithelium led to enhanced gene expression of Sprr2a/b and KRT8 which formed a plug to block sweat gland secretion ducts[26]. However, similar to Lay et al’ s report[11], this article by Cui et al did not show FOXC1 gene expression in mouse epidermis. Comparing our data generated from human primary KC and human skin biopsies with data from Lay et al and Cui et al using murine models, we think FOXC1 may function differently in human epidermis from mouse epidermis.

Although FOXC1 over-expression is found in some types of breast cancer cells, liver cancer cells, acute myeloid leukemia, and increased FOXC1 in these cancer cells is associated with proliferation [27–29], there is no study reported FOXC1 over-expression in keratinocyte-derived cancer. A previous study found that cervical cancer derived cell line, Hela cells, lost FOXC1 gene expression due to gene mutation. When reintroduction of FOXC1 into Hela cells restores the cell growth inhibition [30]. The contradictory results obtained from different cancer cells and primary normal human KC suggest that FOXC1 has pleiotropic functions.

We show here the hierarchy position of FOXC1 in the transcription factor network regulating epidermal differentiation. Recently, a few studies began to study the transcriptional regulatory network for KC terminal differentiation. Transcription factor p63 is a master regulator that is required both for the development and homeostasis of stratified epithelial tissues[31]. ZNF750 is a p63 target gene. It is induced during the progression of KC differentiation, and binds to another KC terminal differentiation transcription factor KLF4. The research results from Sen et al suggested that ZNF750 worked up-stream of KLF4 [5]. They therefore concluded that ZNF750 is a p63 target gene that induces KLF4 to drive terminal epidermal differentiation. A more recent study found that KLF4, ZNF750 and GRHL3 are not the only targets of p63, but also down-stream of MAF and MAFB [9]. In our study, through multiple siRNA silencing experiments, we found that KLF4 and ZNF750 are regulating their expression reciprocally. FOXC1 is the target gene for both KLF4 and ZNF750. To validate our data, we used ChIP assay to analyze whether ZNF750 and KLF4 directly bind to the promoter region of FOXC1. Our results confirmed that ZNF750 and KLF4 indeed bind to -873bp to -643 bp and -663bp to -493 bp upstream of the start codon of FOXC1 (Fig 6). Furthermore, we define that GRHL3 is the target gene of FOXC1, therefore silencing any one of ZNF750, KLF4 and FOXC1 resulted the reduced gene expression of GRHL3. Indeed, the sets of target genes of ZNF750, KLF4, FOXC1 and GRHL3 are overlapped, supporting the assumption that these genes organize as a hierarchy network.

In summary, our work supports a previously unappreciated role for FOXC1 in promoting the human epidermal KC terminal differentiation program; it acts down-stream of ZNF750 and KLF4 and up-stream of GRHL3.

## Materials and Methods

### Human normal KC culture and differentiation

Primary human neonatal foreskin KC from 6 different donors were purchased from Thermo Fisher Scientific and maintained in EpiLife Medium containing 0.06 mM CaCl_2_ and S7 supplemental reagent under standard tissue culture conditions. The cells were seeded in 24 well-dishes at 2x10^5^/well to form a confluent monolayer. The following day, the cells were subjected to differentiation by increasing CaCl_2_ to 1.3mM in the culture media. The cells were harvested for RNA extraction before differentiation, differentiation for 24 hours (D1), 48 hours (D2), 72 hours (D3), 96 hours (D4) and 120 hours (D5). Then RNA and protein were prepared from the cells.

### siRNA knockdown gene expression

FOXC1 siRNA duplexes, ZNF750 siRNA duplexes, KLF4 siRNA and control non-targeting scrambled siRNA duplexes were purchased from Life Technologies. The sequence for KLF4 siRNA are: sense: UGACCAGGCACUACCGUAAtt; antisense: UUACGGUAGUGCCUGGUCAtt. The sequence for ZNF750 siRNA are: sense: CCUCAAUGUUGUGAACGGAtt; antisense: UCCGUUCACAACAUUGAGGct. The sequence for FOXC1 siRNA are: sense 1: GAAAGUCGCUUUCUUUUUAtt; antisense: UAAAAAGAAAGCGACUUUCat. Sense 2: ACUCUCCAGUGAACGGGAAtt, antisense: UUCCCGUUCACUGGAGAGUtg; Sense 3: AGAGGAUCGGCUUGAACAAtt; anti-sense: UUGUUCAAGCCGAUCCUCUgt. KCs were plated in 24 well plates at 1x10^5^ per well the day before transfection. Cells were transfected with siRNA duplexes at final concentration of 10 nM using lipofectamine 2000 according to the manufacturer’s instructions (Invitrogen, Carlsbad, CA). After 24 hours incubation, the cells were replaced with EpiLife supplemented either with 0.06mM or 1.3 mM calcium for up to 120 hours. Then the cells were used for total RNA and protein extraction.

### Total RNA extraction and real-time PCR

Total RNA was extracted using RNeasy mini kit according to the manufacture’s guideline (QIAGEN, MD). RNA was then reverse transcribed into cDNA using superScript® III reverse transcriptase from Invitrogen (Portland, OR) and analyzed by real time RT-PCR using an ABI Prism 7000 sequence detector (Applied Biosystems, Foster City, CA) as previously described(Nomura *et al*, 2003). Primers and probes for human *FOXC1* (Hs00559473_s1), *FLG* (Hs00856927_g1), *IVL* (Hs00846307_s1), *LOR* (Hs01894962_s1), *ZNF750* (Hs01563263_g1), *KLF4* (Hs00358836_m1), *GRHL3* (Hs00297962_m1) and *18S* (Hs99999901_s1) were purchased from Applied Biosystems (Foster City, CA). Quantities of all target genes in test samples were normalized to the corresponding 18S levels.

### Western-blot protein expression

Cells were lysed in protein lysis buffer (20mM Tris-HCl pH7.4, 150 mM NaCl, 1mM EDTA, 1% Triton-X100) supplemented with protease inhibitors. After collecting the aqueous soluble protein, the un-soluble portion were solubilized in 6M urea supplemented with proteinase inhibitors. The proteins were then used for western-blot assay following the standard procedure. The antibodies against human FOXC1, IVL, LOR, FLG, DSC1 and cyclin D were purchased from Abcam (Cambridge, MA). The mouse monoclonal antibody against human β-actin was purchased from Sigma Aldrich (St. Louis, MO).

### Immunohistochemistry staining of normal human skin

Normal human skin were obtained as discarded materials from plastic surgeries after approval of the human research committee of the first affiliated hospital of Jinan University at Guangzhou, China. Written informed consents were obtained from participating human subjects. Rabbit polyclonal anti-FOXC1 antibodies (recognize the region of amino acids 250–300 of FOXC1 protein) were purchased from Abcam (Catalog number ab115201, Cambridge, MA). After sequential steps of deparafinization, antigen retrieval, and inactivation of endogenous peroxidase, normal human skin sections (5 um) were blocked with 5% BSA, then incubated with primary antibody at 40C overnight. The following day, skin sections were washed by 1xPBS, incubated with secondary antibody conjugated HRP for 30 minutes, and then developed with 3, 3’-Diaminobenzidine. Hematoxylin solution was used to stain the cell nucleus. Pictures were acquired using a Leica DM6000 microscope system.

### Overexpression of FOXC1 in undifferentiated primary KC

pLenti-C-RFP and pCMV6-entry-FOXC1(RC215629) plasmids were purchased from Origene Technologies (Rockville, MD). Full-length cDNA of FOXC1 was cut by Sgf I and Mlu I from pCMV6-entry-FOXC1 plasmid and then inserted into pLenti-C-RFP backbone to construct pLenti-C-RFP-FOXC1. The lentiviral particles of pLenti-C-RFP and pLenti-C-RFP-FOXC1 were generated in 293FT cells by co-transfection with packaging plasmids using Lenti-vpak packing kit (Origene Technologies, Rockville, MD). The viral supernatants were harvested and infected undifferentiated primary KC. 5 days after infection, the cells were harvested for RNA extraction and real-PCR.

### RNA-Sequencing of transcriptome profiling

Total RNA were extracted KC transfected with scrambled siRNA and FOXC1 siRNA duplexes under undifferentiated conditions and differentiated for 5 days using RNeasy mini kit (QIAGEN, MD). RNA purity and concentration was measured on an Agilent Bioanalyzer (Agilent Technol., Palo Alto, CA). A total of 1 μg total RNA was used to prepare the Illumina HiSeq libraries according to manufacturer’s instructions of the TruSeq RNA kit. Library preparation and HiSeq sequencing was performed at BGI technologies in China (Shenzhen, China).

Transcript levels were quantified in reads per kilobase of exon per million mapped reads (RPKM). The RPKM reflects the molar concentration of a transcript in the starting sample by normalizing for gene length and for the total read number in the measurement. This allows comparison of transcript levels both within and between experiments. The sequencing reads were analyzed using the computational pipeline of Bowtie, Tophat, and Cufflinks [32]. The mapped and aligned files of this study, along with processed data of RPKM for all detected transcripts have been deposited in the NCBI Gene Expression Omnibus (Access number: GSE76647).

### Chromatin immunoprecipitation (ChIP) assay

ChIP assays were performed using Pierce Agarose ChIP kit (Thermo Scientific, prod#26156)

Following the manufacture’s protocol. Briefly, human primary KC were differentiated by elevated the calcium to 1.3mM and cultured for 3 days. The cells were then incubated with 1% formaldehyde for 10 minutes at 37°C, followed by incubation with 1x Glycine solution for 5 minutes in room temperature. The cells were then collected, lysed and digested by MNase. Protein-chromatin complexes were immunoprecipitated with antibodies against KLF4, ZNF750, or rabbit IgG (negative control). Anti-KLF4, ZNF750 and rabbit IgG were purchased from Abcam (Cambridge, MA). Complexes were separated by incubation with ChIP grade proteinA/G agarose. Chromatin DNA fragments were then eluted and purified for quantitative real-time PCR. The primers used for amplification of specific regions of FOXC1 promoter were described in supplemental materials.

### Statistical and Bioinformatic Analysis

Partek Genomics Suite (www.partek.com) was used to calculate the normalized gene expression levels and run the statistical analysis on the RNA-seq data. Partek’s transcript algorithm is similar to the one in Xing et al [33] except Partek quantifies expression across the whole genome at the same time rather than each gene separately, and accounts for the fragmentation step in RNA-seq by normalizing by transcript length (http://www.partek.com/Tutorials/microarray/User_Guides/RNASEQ.pdf). A two-way ANOVA model was applied to the experimental groups and corrected for multiple testing at FDR<0.01. The statistical analysis of real-time qPCR data was conducted using Graph Pad prism, version 5.03 (San Diego, CA). Comparisons of expression levels were performed using ANOVA techniques and independent sample *t* tests as appropriate. Differences were considered significant at *P*<0.05.

## Supporting Information

S1 Appendix Supplemental materials and methods. (DOCX)

S1 Fig Cell cycle analysis of KC transfected with scrambled siRNA and FOXC1 siRNA duplexes. (PDF)

S1 Table The top 10 most up-regulated TFs during the course of KC differentiation. (DOCX)

## Abbreviations

ChIP: Chromatin immune-precipitation
DSC1: Desmocollin 1
FLG: Filaggrin
FOXC1: Forkhead box C1
IVL: Involucrin
KC: Keratinocytes
KLK: Kallikrein
KRT: Keratin
LCE: Late cornified envelope
LOR: Loricrin
RNA-seq: RNA-sequencing
RPKM: Reads Per Kilobase of transcript per Million mapped reads
siRNA: Small interference RNA
UD: Un-differentiated keratinocytes
