# Correction: Forkhead Box C1 Regulates Human Primary Keratinocyte Terminal Differentiation

**DOI:** 10.1371/journal.pone.0191127

**Published:** 2018-01-05

**Authors:** Lianghua Bin, Liehua Deng, Hengwen Yang, Leqing Zhu, Xiao Wang, Michael G. Edwards, Brittany Richers, Donald Y. M. Leung

In the Funding section, the grant number from the funder National Natural Science Foundation of China is listed incorrectly. The correct grant number is: 81371716 (LB).
