# Supplementary material for: Forkhead Box C1 Regulates Human Primary Keratinocyte Terminal Differentiation
Source: PLoS One. 2016 Dec 1;11(12):e0167392. doi: 10.1371/journal.pone.0167392 (PMC5132327; doi:10.1371/journal.pone.0167392)
Supplement: S1 Appendix — (DOCX) [file pone.0167392.s001.docx]

**S1 Appendix**

**Supplemental Materials and Methods:**

1. **RNA-sequencing profiling for human Normal KC differentiation model.**

Normal human KC were purchased from Invitrogen (Portland, OR) and maintained in EpiLife Medium containing 0.06 mM CaCl_2_ and S7 supplemental reagent (Invitrogen) under standard tissue culture conditions. The cells were seeded in 6 well-dishes at 1x10^6^/well to form a confluent monolayer. The following day, the cells were subjected to differentiation by increasing CaCl_2_ to 1.3mM in the culture media. The cells were harvested for RNA extraction before differentiation, differentiation for 24 hours, 48 hours, 72 hours, 96 hours and 120 hours.

Total RNA was extracted using QIAzol®Lysis reagent (QIAGEN, MD). In brief, cells were lysed in 1ml of QIAzol®Lysis reagent, then 200 ul chloroform was added to the lysate, mixed well, and centrifuged at 7500g for 5 minutes at 4^0^C. The aqueous phase was transferred to a new tube and 500 ul of isopropanol was added, mixed well and incubated for 10 minutes, then centrifuged at 12,000g for 15 minutes at 4^0^C. The RNA pellet was washed twice using 75% of ethanol. After the pellet was dried, the total RNA was dissolved in 50 ul of RNase-free water and stored in -80^0^C.

RNA purity and concentration was measured on an Agilent Bioanalyzer (Agilent Technol., Palo Alto, CA). A total of 5 µg total RNA was treated with Ribo-Zero^TM^  Magnetic Kit (Epicentre) to deplete ribosome RNA (rRNA). After rRNA depletion, the RNA was used to prepare the Illumina HiSeq libraries according to manufacturer’s instructions of the TruSeq RNA kit. Library preparation and HiSeq sequencing was performed at BGI technologies in China (Shenzhen, China).

The RNA-seq sequencing reads were analyzed using the computational pipeline of Bowtie, Tophat, and Cufflinks. The mapped and aligned files of this study, along with processed data of RPKM for all detected transcripts have been deposited in the NCBI Gene Expression Omnibus (<http://www.ncbi.nlm.gov/geo/query/acc.cgi?acc=GSE73305>). All transcription factors collected in the National Center of Biotechnology information data base were used to retrieve transcription factors profile that were expressed in our RNA-seq data set. A total of 1718 transcription factors

were detected in this data-set. The top 10 most up-regulated TFs were shown in Table S1

1. **Cell cycle analyses of scrambled and FOXC1 siRNA silenced undifferentiated keratinocytes.**

Human primary KC were seeded in six well dishes the day before transfection of siRNA

duplexes. The following day, the cells were transfected with scrambled siRNA and FOXC1 siRNA using lipofectamine 2000 according to the manufacture’s guideline. Two days after transfection, the cells were detached from the culture dishes using 0.05% of trypsin digestion. The cells were then collected, washed by cold 1xPBS buffer and fixed in 70% ethanol for 2 hours in 4^0^C. The cells were then washed by cold 1xPBS twice, then resuspended cells in 500 µl propodium iodide /Triton X-100 staining solution(10 ml of 0. 1 % (v/v) Triton X-100 (Sigma) in PBS add 2 mg DNAse-free RNAse A (Sigma) and 0.40 ml of 500 µg/ml propodium iodide ( Roche)), and incubated the cells for 15 minutes in 37^0^C. The data were acquired on a BD FACSCalibur Flow Cytometer. The data were analyzed using FlowJo V10.2 program.

1. **Primers used for ChIP assays were purchased from Integrated DNA Technologies.**

The sequences as below which covered the up-stream 1974 bp region of FOXC1 start code.

FOXC1-1F: 5’ ggagctgttaaatgcgtcac 3’; FOXC1-1R: 5’ ttcgccgtcgctgggtggtt 3’; FOXC1-2F: 5’ aaccacccagcgacggcgaa 3’; FOXC1-2R: 5’ cggtgacattagcaggtgtg 3’; FOXC1-3F: 5’ cacacctgctaatgtcaccg 3’; FOXC1-3R: 5’ ccacttggaggttctcctgt 3’;

FOXC1-4F: 5’ acaggagaacctccaagtgg 3’; FOXC1-4R: 5’ cgttcgcgcagacttcctgg 3’; FOXC1-5F: 5’ ccaggaagtctgcgcgaacg 3’; FOXC1-5R: 5’ aatgagccggaggccgagcc 3’; FOXC1-6F: 5’ ggctcggcctccggctcatt 3’; FOXC1-6R: 5’ agccgcttaaggaagcattg 3’; FOXC1-7F: 5’ caatgcttccttaagcggct 3’; FOXC1-7R: 5’ agaccgccttgcaggaactc 3’;

FOXC1-8F: 5’ gagttcctgcaaggcggtct 3’; FOXC1-8R: 5’ tgcgctgtgcgtccggctgc 3’; FOXC1-9F: 5’ gcagccggacgcacagcgca 3’; FOXC1-9R: 5’ gctggacacggagtagcgcg 3’
