# Supplementary material for: Forkhead Box C1 Regulates Human Primary Keratinocyte Terminal Differentiation
Source: PLoS One. 2016 Dec 1;11(12):e0167392. doi: 10.1371/journal.pone.0167392 (PMC5132327; doi:10.1371/journal.pone.0167392)
Supplement: S1 Fig — (PDF) [file pone.0167392.s002.pdf]

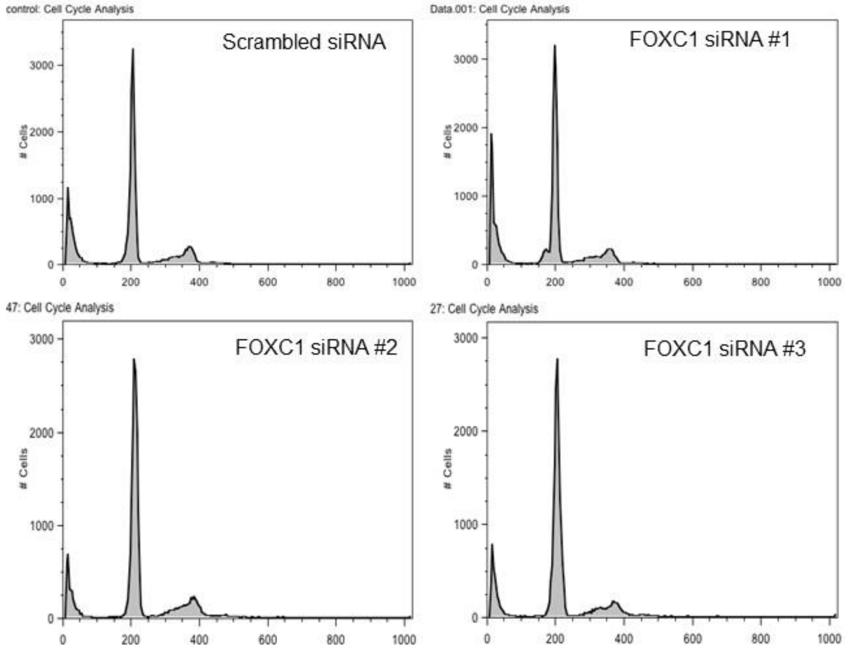

S1 Fig. Knock-down FOXC1 in undifferentiated KC doesn't change cell cycle parameter as compared to cells transfected with scrambled siRNA duplexes.
