# Supplementary material for: Forkhead Box C1 Regulates Human Primary Keratinocyte Terminal Differentiation
Source: PLoS One. 2016 Dec 1;11(12):e0167392. doi: 10.1371/journal.pone.0167392 (PMC5132327; doi:10.1371/journal.pone.0167392)
Supplement: S1 Table — (DOCX) [file pone.0167392.s003.docx]

| S1 Table. The top 10 most up-regulated TFs during the course of KC differentiation | | | | | | | |  |
| --- | --- | --- | --- | --- | --- | --- | --- | --- |
| Symbol | UD (RPKM) | D1 (RPKM) | D2 (RPKM) | D3 (RPKM) | D4 (RPKM) | D5 (RPKM) | D5/UD (Fold) | KC differentiation related |
| FOXN4 | 0 | 0 | 0 | 0 | 0 | 0.5412 | #DIV/0! | Unknown |
| EGR3 | 0.0005 | 0.0005 | 1.0698 | 3.9276 | 3.6454 | 4.2583 | 8832.1580 | Yes |
| ID4 | 0.3861 | 2.8649 | 1.5560 | 29.9262 | 49.6678 | 127.5524 | 330.3753 | Yes |
| ELF3 | 0.0281 | 0.2368 | 4.6109 | 0.9015 | 0.8191 | 2.1767 | 77.3462 | Yes |
| MKX | 0.0123 | 0.4386 | 0.7310 | 0.1232 | 0.3726 | 0.9201 | 75.0049 | Unknown |
| MSC | 0.0666 | 0.5373 | 1.1084 | 0.6762 | 3.8043 | 3.6151 | 54.3087 | Unknown |
| EGR2 | 0.0476 | 0.2170 | 0.5285 | 1.2632 | 2.5557 | 1.9719 | 41.4323 | Yes |
| POU2F3 | 0.8130 | 1.4098 | 5.6734 | 14.2372 | 9.7706 | 25.6934 | 31.6046 | Yes |
| NFE2 | 0.0269 | 0.2553 | 0.0613 | 0.1359 | 1.5390 | 0.7889 | 29.3715 | Unknown |
| FOXC1 | 6.4787 | 12.9929 | 15.2401 | 18.7230 | 73.3763 | 188.3834 | 29.0775 | Unknown |
